# Precision medicine approaches in obstructive sleep apnoea: The role of dentist–sleep physician partnerships

**DOI:** 10.1111/adj.13039

**Published:** 2024-10-01

**Authors:** GM Stewart, BK Tong, PA Cistulli

**Affiliations:** ^1^ Charles Perkins Centre and Sydney Medical School University of Sydney Camperdown Sydney Australia; ^2^ Department of Respiratory and Sleep Medicine Royal North Shore Hospital St Leonards Sydney Australia

**Keywords:** Patient care, oral appliance therapy, risk factors, mandibular advancement splints, predicting treatment outcomes

## Abstract

Obstructive Sleep Apnoea (OSA) is a common heterogenous sleep disorder that is associated with a wide range of comorbidities and consequences, including the development of neurocognitive and cardiometabolic disorders. The heterogeneity of OSA necessitates a precision medicine approach to accurately diagnose this condition and to effectively manage patients. One of the primary models of precision medicine is described by the P4 approach of *predicting* those who are susceptible to disease, *preventing* the occurrence of disease, *personalizing* treatment, and encouraging patients to *participate* in their individual healthcare journey. Recent advances in oral appliance therapy and OSA monitoring techniques have fostered an exciting opportunity for enhanced collaboration between dentists and sleep physicians to optimize OSA precision medicine care. This review aims to discuss the sources of heterogeneity among OSA patients, provide an overview of the growing applications of oral appliance therapy and tailored monitoring programs for OSA that are shifting treatment to a more personalized and participatory model of care, and outline the pivotal role of dentists in managing patients with OSA.

**CLINICAL RELEVANCE**

Recent advances in oral appliance therapy, alongside a growing understanding that obstructive sleep apnoea (OSA) is a common heterogeneous condition, have fostered an exciting opportunity for enhanced collaboration between dentists and sleep physicians. By embracing precision medicine approaches in OSA, the discipline of dental sleep medicine can support a model of care that improves diagnostic accuracy, enhances therapeutic options, and optimizes patient outcomes. This shift in clinical practice is of high relevance to dentists, who can play a pivotal role in the diagnosis and management of OSA.

**Abbreviations and Acronyms**
AHI: apnoea‐hypopnoea index
ESS: epworth Sleepiness Scale
MAS: mandibular advancement splint
OSA: obstructive sleep apnoea
PAP: positive airway pressure
PSG: polysomnogram
REM: rapid eye movement

## INTRODUCTION

Precision medicine is a healthcare approach that aims to recognize and treat unique disease characteristics by providing targeted and individualized therapies that optimize health outcomes. The successful application of precision medicine techniques across several fields of medicine and chronic diseases, such as oncology and heart failure,^1^, ^2^ has highlighted the benefits of refining disease diagnosis and management in accordance with distinctive disease subtypes and traits.^3^ While several modes of precision medicine have been developed,^4^ the P4 approach, initially described by Hood and colleagues,^5^ is a conceptualization of a healthcare model that is based on four key principals that make medicine more *predictive*, *preventive*, *personalized*, and *participatory*. The P4 model has emerged from three broader trends in medicine that include (1) an increasing ability to decipher and intervene in the complex biology of disease; (2) a growing capacity to collect, integrate, and analyse large and complex sets of data; and (3) rising consumer interest in and access to the management of their own health. In this regard, the discipline of dental sleep medicine – that is, the study and treatment of disorders that impact sleep quality such as snoring, sleep apnoea, bruxism, xerostomia, hypersalivation, gastro‐oesophageal reflux, and orofacial pain^6^, ^7^ – is well poised to adopt precision medicine approaches that improve individuals' health outcomes.

Obstructive Sleep Apnoea (OSA) is a common sleep disorder characterized by repetitive airway collapse leading to intermittent oxygen desaturations, fragmented sleep, and exaggerated intra‐thoracic pressure swings.^8^ The worldwide prevalence of OSA is estimated to be as high as 1 in 8 individuals – that is, ~1 billion persons^9^ – and is associated with a wide range of comorbidities and consequences, including neurocognitive and cardiometabolic disorders, occupational and vehicle accidents, and economic loss.^10^, ^11^, ^12^, ^13^ Despite the high prevalence, OSA is increasingly recognized as a highly heterogenous condition that is associated with a variety of underlying causes, varying expression of disease, and a range of poor outcomes that require a precision medicine framework to ensure effective long‐term management and optimize patient outcomes.^14^, ^15^, ^16^ In this regard, dental practitioners play a key role in enhancing OSA precision medicine approaches by identifying and supporting OSA patients who are most likely to respond well to dental interventions such as oral appliance therapy.^3^, ^6^ This review provides an overview of current challenges and opportunities for precision medicine approaches in OSA, with specific focus on the heterogeneity of OSA phenotypes and the application of oral appliance therapy in clinical practice. Specifically, this review will (1) describe the traits, risk factors, and pathophysiology of OSA that aid in the prediction and prevention of this condition; (2) outline the growing application of oral appliance therapy and tailored monitoring programs that are shifting OSA treatment to a more personalized and participatory model of care; and (3) describe the pivotal role of dentists in screening for OSA and managing patients who opt for oral appliance therapy.

## CURRENT CHALLENGES IN PREDICTING AND PREVENTING OSA

The presence and severity of OSA are broadly defined by the number of times per hour of sleep that the airway completely (Apnoea) or partially (Hypopnoea) collapses, triggering a drop in blood oxygenation levels, termed the apnoea–hypopnoea index (AHI). The AHI is most often determined via an overnight sleep study or polysomnogram (PSG) and used clinically to distinguish the presence and severity of OSA and guide therapeutic intervention. However, the current clinical thresholds used to define the presence of OSA (AHI ≥5 events/h), and to demarcate the severity of OSA as mild (AHI 5–14.9 events/h), moderate (AHI 15–29.9 events/h), or severe (AHI ≥30 events/h) are largely arbitrary and do not correlate well with symptoms, comorbidities, or treatment outcomes.^17^ The frequency and magnitude of oxygen desaturations that occur alongside apnoea–hypopnoea events, often characterized by the Oxygen Desaturation Index, (ODI) and the mean oxygen saturation and nadir during sleep are additional clinical markers used to demarcate OSA severity, again with varying degrees of prognostic accuracy.^18^ There is growing recognition that the AHI‐ and ODI‐based criteria for grading the presence and severity of OSA are not an ideal definition of the disease, and as such, the search for novel metrics that better classify OSA is an ongoing priority.^17^ Despite the limitations of AHI‐ and ODI‐based criteria, adequate treatment of moderate‐to‐severe OSA is associated with major improvements in both OSA symptoms and clinical outcomes,^19^, ^20^ and untreated OSA is associated with serious long‐term adverse outcomes.^21^ However, symptomatic and clinical improvements are not universally observed following OSA treatment,^22^, ^23^ due in part to inadequate or ineffective treatment strategies that are not based on individual needs and preferences.^23^ In this regard, accurate identification and understanding of distinct clinical OSA phenotypes and downstream pathophysiological consequences are essential for optimizing OSA treatment and delivering an effective personalized medicine approach.^23^

### Characteristics and traits of OSA

**Fig. 1:**
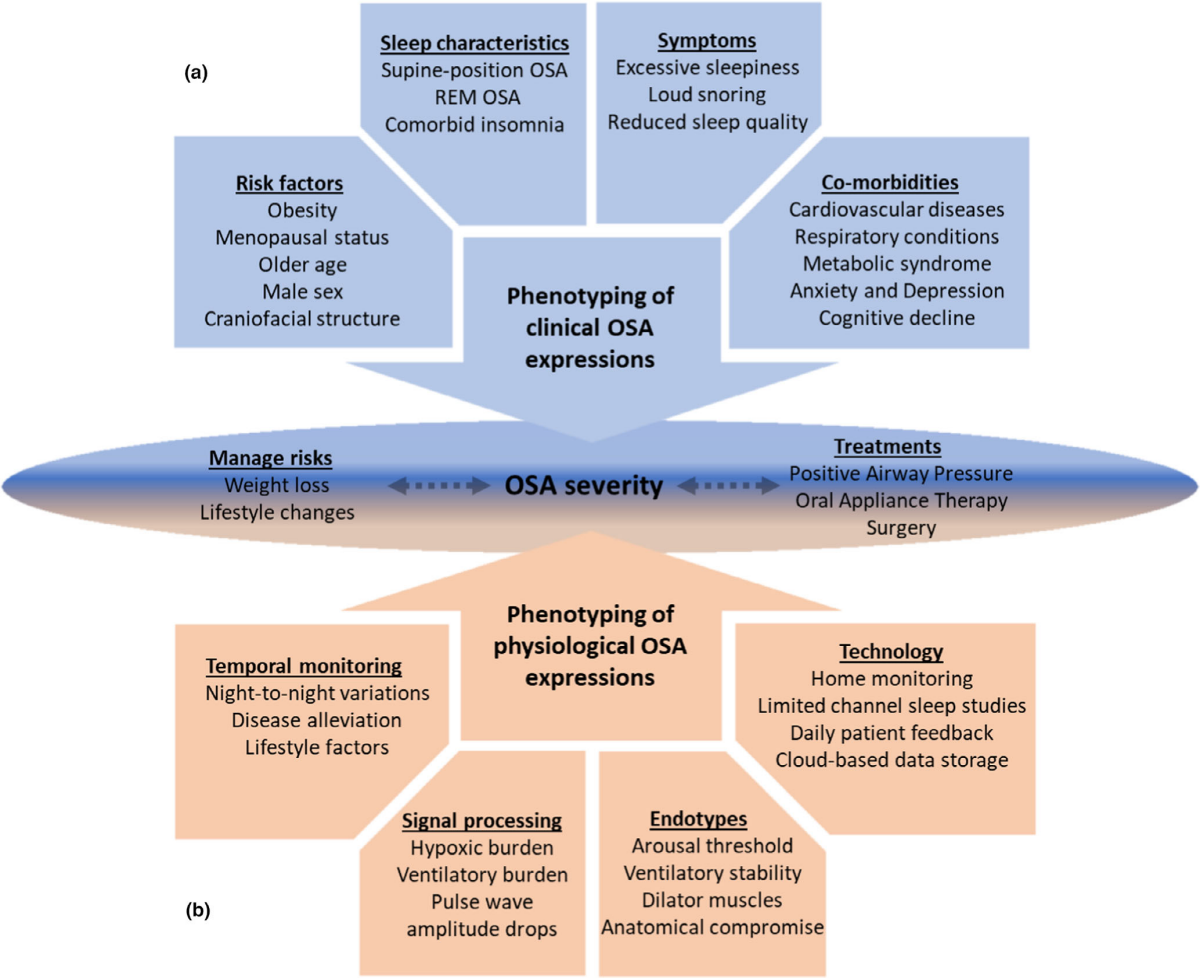
Schematic of the clinical (a) and physiological (b) features typically used to define the phenotypic expressions of obstructive sleep apnoea. OSA, obstructive sleep apnoea; REM, rapid eye movement.

The typical features used to define the phenotypic expressions of OSA include age, sex, anatomical characteristics, sleep posture and sleep stage, symptomology, and comorbidities.^24^ These traits can be broadly grouped into risk factors for developing OSA, sleep characteristics of OSA, symptoms of OSA, and comorbidities that often have bidirectional relationships with OSA (Fig. 1a). In addition to these clinical OSA phenotypes, various new tools and techniques to help further characterize the pathophysiological consequences of OSA are currently undergoing clinical research and validation.^25^ These research tools, such as signal processing techniques to quantify the hypoxic burden associated with OSA and home‐based monitoring devices that track night‐to‐night OSA fluctuations, are helping to further refine OSA diagnoses and inform individualized treatment approaches (Fig. 1b).

#### Risk factors for OSA

The presence and severity of OSA are highly associated with biological factors such as older age, male sex, excess body weight, and craniofacial abnormalities.^26^, ^27^ While age, biological sex, and skeletal structure are relatively fixed OSA traits, obesity is a major modifiable public health problem that has a high and rising prevalence.^28^ A large proportion (~70%) of patients diagnosed with OSA are overweight or obese, and weight gain is associated with worsening OSA and weight reduction with improvements in OSA.^29^ The relationship between excess weight and OSA is complex and has broad effects beyond increased adiposity of the pharynx region. For example, by reducing functional lung volumes and inducing small airway dysfunction, excess body weight can increase the propensity for airway collapse and worsen hypoxaemia in patients with OSA.^28^, ^30^ The prevalence of OSA rises markedly in older women and after menopause, reaching as high as ~50%–70% in postmenopausal women.^31^ Craniofacial structure and anatomical variations are also key predisposing features of OSA, whereby conditions that compromise the pharyngeal airway space contribute to OSA.^32^ Indeed, the narrower the upper airway – whether a result of micrognathia, retrognathia, or other pharyngeal musculoskeletal abnormalities – the greater the propensity for airway collapse and thus the presence and severity of OSA.^27^ Grading of the oropharyngeal opening with physical examination scales, such as the Mallampati scoring system, can indicate risk of OSA; however, such scales do not correlate well with OSA severity.^33^ In this regard, the profiling of OSA risk factors and the initiation of first‐line treatments (such as positive airway pressure, or PAP) should be managed in relation to both modifiable and unmodifiable anatomical predispositions.

#### Sleep‐related characteristics of OSA

During the clinical diagnosis of OSA, sleep posture and sleep stage‐related OSA are two of the most common clinical OSA phenotypes. Supine position‐related OSA, where apnoea–hypopnoea episodes predominantly occur while in a supine position and are infrequent or absent while in a prone or lateral position, has a prevalence of up to 50% of all OSA diagnoses.^34^, ^35^ Interestingly, individuals with supine‐dependent OSA are more likely to be younger and less obese with a smaller neck circumference and have fewer symptoms and comorbidities. While positional therapy devices that restrict supine sleeping are an option for patients with supine‐dependent OSA, PAP remains the most common treatment. Apnoea–hypopnoea episodes can also transpire throughout the different sleep stages, including during both rapid eye movement (REM) and non‐REM stages of sleep, or be limited to REM only. During REM sleep, the airway is most prone to collapse due to a reduced afferent muscle tone (atonia), and patients with REM‐specific OSA might be at a heightened risk of developing cardiovascular diseases.^36^

#### Symptoms of OSA

Two major side effects and predictors of OSA are loud and persistent snoring accompanied with air ‘gasping’ (often identified by a bed partner) and excessive daytime sleepiness. Daytime sleepiness is a hallmark complaint of untreated OSA that is associated with poorer health outcomes and elevated rates of occupational and motor vehicle accidents.^37^ The Epworth Sleepiness Scale (ESS) is a simple and accessible questionnaire often used to gauge daytime sleepiness, although it lacks specificity as a diagnostic tool for OSA. While excessive daytime sleepiness (defined by an ESS score of >9) is considered a major clinical sequalae of OSA, its prevalence in the general OSA population varies from 50% to 75%. Ongoing attempts to better identify individuals with key clusters of OSA symptoms, called symptom subtypes, are being developed and might help guide individual treatment preferences and responses.^38^ For example, sleepy OSA patients are at much higher risk of developing cardiovascular disease and have unique features to non‐sleepy OSA patients during polysomnography, such as lower sleep latency and higher ODI.^39^ Effective PAP treatment in sleepy OSA patients is also associated with greater benefits than in non‐sleepy OSA patients.^40^

#### Co‐morbidities associated with OSA

A major challenge for precision medicine approaches in OSA is identifying and managing the presence of comorbidities,^41^ which might have bidirectional relationships with OSA. OSA patients have very high rates of comorbid cardiovascular diseases such as systemic hypertension and coronary artery disease,^12^ respiratory diseases such as COPD,^42^ and metabolic disorders such as diabetes mellitus and dyslipidaemia.^11^ In addition, a high proportion of OSA patients suffer from comorbid insomnia, and there are high rates of anxiety, depression, and neurocognitive impairment in OSA patients.^13^ The presence of these conditions in OSA patients is strongly associated with prognosis and mortality risk.^43^ Given that a high comorbidity burden worsens prognosis, and new conditions significantly associated with OSA prognosis are increasingly reported, the role of comorbidities in OSA treatment is emerging as a key focus.^43^ Indeed, some data suggest that OSA treatment might be especially protective in patients with comorbidities, such as by reducing OSA burden *and* lowering blood pressure in hypertensive OSA patients.^28^ However, low adherence to first‐line treatments such as PAP remains a major issue. Therefore, the management of OSA requires multi‐disciplinary care that effectively manages both OSA *and* any associated comorbid conditions in a manner that is well accepted by the patient.

### Evolving techniques to understand OSA pathophysiology

#### Simplified testing methods

While methods to quantify OSA symptoms and biological traits such as weight and craniofacial features associated with OSA are widely accessible, sleep‐related phenotypes of OSA require time‐ and resource‐heavy clinical assessments that are not easily scaled up to meet the growing prevalence of OSA. Home‐based portable sleep recording devices that are somewhat comparable to the gold standard laboratory‐based ‘level 1’ polysomnography (PSG) are acceptable for the diagnosis of moderate to severe OSA and are helping to alleviate the long waitlists for lab‐based sleep studies.^44^ The development of ‘level 3’ and ‘level 4’ limited channel sleep recording devices, such as pulse oximetry‐derived pulse signal analysers and under‐mattress pneumatic analysers,^45^ is enabling more cost‐ and time‐effective alternatives for identifying and monitoring OSA presence and severity. Numerous devices are currently undergoing validation studies and being implemented across clinical environments, albeit with varying degrees of accuracy and reliability.^45^, ^46^

#### Temporal monitoring of OSA

The development of less intrusive sleep monitoring devices has enabled multi‐night testing at home and identified high rates of night‐to‐night variability in the presence and severity of OSA.^47^, ^48^ Assessing OSA across multiple nights might lead to more accurate diagnoses and enhance patient monitoring during critical periods such as the initiation of new therapies. Furthermore, the impact of lifestyle factors such as physical activity patterns and alcohol consumption on OSA can be tracked if temporal monitoring of sleep habits and OSA severity are available. Multi‐night sleep monitoring devices can also provide daily reports (via apps and cloud‐based data storage), which can encourage patient engagement and enable health professionals to assess the effectiveness of targeted interventions or treatments.

#### Advanced signal processing of polysomnography recordings

Alternative novel markers of OSA severity and the downstream physiological consequences of OSA are also being derived from the physiological signals acquired during sleep studies in clinical research trials. For example, quantitative measures of the total overnight ventilatory effort, or ‘ventilatory burden’^49^ and total overnight nocturnal hypoxaemia, or ‘hypoxic burden’^50^ have recently been developed and are helping overcome some of the limitations of the AHI and ODI‐based criteria.

#### Novel pathophysiological features of OSA

Methods to classify the pathophysiological traits that cause and/or exacerbate OSA, often termed ‘endotypes’, are also undergoing investigation to determine their applicability for clinical implementation.^25^, ^51^ The currently accepted endotypes of OSA include unstable ventilatory control (i.e., increased loop gain), low arousal threshold (i.e., increased propensity to awakening), pharyngeal dilator muscle dysfunction, and anatomical compromise of the upper airway.^25^ These endotypes can be determined through direct measurement (gold standard) or estimated via mathematical analysis of polysomnography data, of which there are several approaches currently under debate and which require further validation before widespread clinical adoption can be considered.^52^, ^53^

#### Technology driven precision medicine in OSA

Digital technologies are an important enabler of precision medicine approaches in OSA. The current and evolving technologies available in sleep medicine have confirmed that OSA is a heterogeneous chronic condition that requires efficient long‐term management. Alongside a growing ability to process large amounts of data and accurately characterize OSA subgroups and phenotypes, the treatment landscape is shifting to one where it is crucial for the method of treatment to be effective and well accepted by the patient. In this regard, it is becoming increasingly clear that OSA therapy cannot be limited to a single strategy, and a multi‐disciplinary approach combined with effective provider‐patient communications and systematic long‐term follow‐up is required to achieve effective and long‐lasting management of OSA.

## PERSONALIZED THERAPY FOR OSA

### The historic ‘one size fits all’ approach to OSA

At present, PAP is the gold standard therapy for OSA^54^ and is routinely prescribed to patients with OSA as a ‘one size fits all’ approach. However, at least thirty percent of patients are non‐adherent to PAP within four weeks of commencing treatment.^55^ Common factors leading to PAP discontinuation include mask intolerance, pressure intolerance, lack of symptomatic benefit, and preference for non‐PAP therapies for OSA.^56^, ^57^, ^58^ Nonetheless, a wealth of studies have showed that consistent and adequate use of PAP in patients with OSA improves neurocognitive and cardiovascular functions and quality of life.^59^, ^60^ However, there is some inter‐individual variability in health benefits among patients using PAP. For example, patients with OSA and minimal symptoms might experience no beneficial impacts on blood pressure despite optimal PAP use.^61^ In contrast, systolic blood pressure can decrease by at least 5 mm Hg in patients with resistant hypertension and OSA on PAP therapy.^62^ Studies have suggested that nocturnal hypertension, circadian blood pressure, and nocturnal heart rate are predictors of blood pressure decrease with PAP therapy.^63^ Moreover, a recent study compared the three common clinical phenotypes of OSA, sleepy, excessively sleepy, and disturbed sleep and the patients' responses to PAP.^64^ Notably, the study showed that adherent PAP users with disturbed sleep reported similar changes in insomnia‐related symptoms as those who were not adherent to PAP. This finding suggests that further intervention is required in this subgroup to address the insomnia‐related symptoms. Furthermore, this highlights the benefit of understanding a patient's phenotype in personalizing therapy for OSA.

### Alternatives to the ‘one size fits all’ approach to OSA

Oral appliance therapy, specifically mandibular advancement splints (MAS), is considered the main alternative to PAP therapy.^65^ MAS passively protrudes the mandible anteriorly to increase the calibre of the upper airway, specifically lateral expansion of the velopharynx and anterior movement of the tongue, thereby increasing soft tissue tone and reducing airway collapsibility.^66^, ^67^ There are a wide range of available MAS devices, and determining patient suitability requires professional screening of dental structures and contraindications (e.g., TMJ disease, periodontal disease). This provides an important role for dentists in the management of OSA in collaboration with medical practitioners. In suitable patients, treatment of OSA with MAS has been shown to improve symptoms of OSA and quality of life similar to PAP.^68^ Moreover, MAS is not inferior to PAP in controlling blood pressure.^69^ Patients are also more adherent to MAS compared to PAP, according to objective compliance data at 1‐year follow‐up.^58^, ^70^ However, only fifty percent of patients using MAS achieve complete resolution of OSA^22^ and prediction of responders to MAS remains elusive.

Clinical predictors of favourable response to MAS include lower BMI, younger age, female sex, low Mallampati classification, and mild severity of OSA.^71^ In addition, craniofacial features such as retrognathism, a larger cranial base angle, and shorter anterior face height are associated with a better response to MAS.^72^ Moreover, supine predominant OSA and REM predominant OSA are associated with incomplete responses to MAS.^73^ However, these clinical and polysomnographic predictors appear to fail in prospective validation studies of treatment responses.^73^, ^74^

Novel endotyping techniques that classify pathophysiological traits of OSA have recently shown that patients with less anatomical deficiency and better ventilatory stability (lower loop gain) are suitable candidates for MAS.^75^ Two studies utilizing these methods to predict MAS response from estimated OSA endotypes showed similar findings of milder anatomical deficiencies, lower loop gain, higher arousal threshold, and weaker upper airway dilator muscle response for greater MAS efficacy.^76^, ^77^ Prospective validation of these endotypes for MAS response remains to be assessed.

Direct visual assessment of upper airway structures using Drug‐Induced Sleep Endoscopy (DISE) to identify suitable patients for MAS has shown good predictive accuracy.^78^ Specific levels of upper airway collapse, including tongue base collapse, have been associated with favourable oral appliance therapy response.^79^ Conversely, concentric collapse of the oropharynx and anteroposterior collapse of the epiglottis is associated with poor oral appliance therapy outcomes.^79^, ^80^ In addition, the method of mandibular advancement manoeuvre during DISE has shown some potential as a predictive tool for MAS response.^81^, ^82^ These findings highlight the utility of collaborating with otolaryngologists in providing multi‐disciplinary care to enhance the delivery of oral appliance therapy for individual patients diagnosed with OSA.

### The role of combination therapy in OSA

Since non‐PAP therapies tend to be partially effective, there is logic in combining such therapies to achieve an additive or synergistic effect, an emerging concept towards personalizing therapy for OSA. For example, combining supine avoidance therapy with MAS,^83^ PAP and MAS,^84^ and MAS and oro‐nasal expiratory positive airway pressure valves^85^ have shown promise in controlling OSA severity. One proof‐of‐concept study showed a model in which targeted combination therapy with MAS that was informed by endotype characterization of OSA reduced OSA severity in 95% of incomplete responders to MAS.^86^ Long‐term compliance and acceptability of combination therapy for OSA among patients remain to be studied. Dentists and sleep physicians will need to work collaboratively during patient care and involve patient participation to leverage the benefits of combination therapy for OSA with an oral appliance.

## PATIENT PARTICIPATION AND PREFERENCE IN OSA MANAGEMENT

Patient participation in their own healthcare management is a key component of precision medicine to optimize treatment adherence and health outcomes. Given that almost all patients with OSA are counselled to improve their overall sleep behaviours and lifestyle habits, either independently or in combination with the initiation of PAP and/or MAS, consideration of a patient's preferences and motivations is crucial for effective treatment. Indeed, treatment pathways that align with a patient's preferences are associated with improved adherence to therapy usage and better health outcomes.^87^

The personalized management of OSA is strategically positioned with technological advancements in OSA therapy devices to allow for feedback to patients about their ongoing care. For example, the growth of remote monitoring and patient engagement platforms for PAP has showed improvements of at least an hour of extra PAP usage per night among patients actively engaged with their daily therapy performance.^88^ Moreover, patient engagement tools that provide coaching, troubleshooting tips, and simple therapy metrics allow for patients to feel engaged and in control of their therapy for OSA, thereby improving therapy adherence.^89^

Similar patient feedback platforms for oral appliances are currently lacking at this stage but are firmly on the horizon. However, some oral appliances now have temperature‐sensing chips, which enable objective tracking of treatment compliance.^90^ Three distinct usage patterns of oral appliance therapy have been identified: consistent users, inconsistent users, and non‐users.^91^ These usage patterns are observed within the first month of therapy initiation, suggesting the potential of early intervention for patients struggling with oral appliance therapy.

Novel approaches to incorporate new sensors into oral appliances are being developed to enable similar patient feedback platforms to PAP therapy. The concept of a smart oral appliance capable of measuring blood oxygen saturation, heart rate and respiratory rate, and sleep stage^92^, ^93^ has the potential to provide new metrics to quantify treatment effectiveness.^94^ These novel approaches have the potential to improve patient selection and individualized data available for personalizing OSA therapy and to assist the treatment team of dentists and physicians to monitor patient progress.

## MULTI‐DISCIPLINARY OSA CARE

**Fig. 2:**
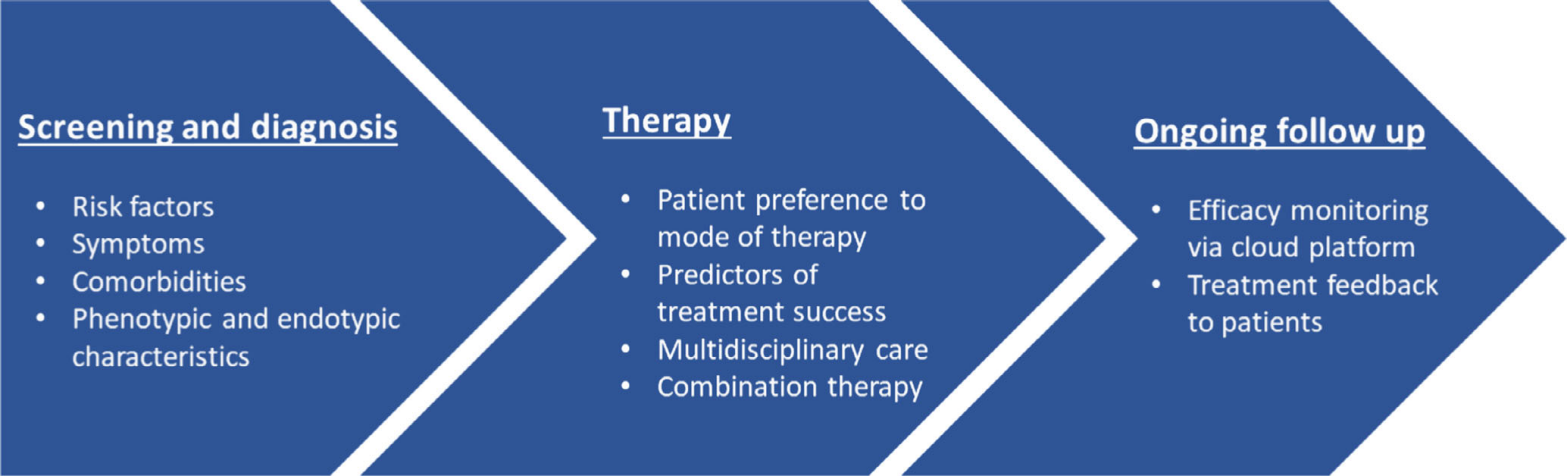
Management of OSA therapy utilizing P4 model of care.

Sleep physicians currently play a primary role in OSA care, from diagnosing, making therapy recommendations, and providing ongoing follow‐up.^93^ This is problematic given the very high prevalence of OSA and the limited number of sleep physicians, highlighting the need to build capacity for OSA care in the primary care setting in the future. Due to the heterogeneous nature of OSA and variable effectiveness in OSA therapies, multi‐disciplinary care incorporating dentists, otolaryngologists, sleep psychologists, and other clinical specialists is essential to enhance prognostic outcomes.^94^, ^95^ The role of dentist‐sleep physician partnerships in the multi‐disciplinary care model is important in both screening for OSA and ongoing management of dental therapies (Fig. 2). For example, dentists might screen patients using the OSA50, STOP‐BANG, and Berlin questionnaires, assess craniofacial characteristics, and degree of airway crowding to determine a patient's likelihood of OSA. When there is a high risk for OSA, dentists can recommend referral to a sleep physician for formal assessment and consultation.^96^ If oral appliance is recommended, screening for suitability can be performed by both dentist and otolaryngologist to understand the site of airway collapse and the probability of treatment success.

The ongoing management of oral appliance therapy requires active participation from the patient, sleep physician, and dentist. This involves overseeing titration of the appliance and assessment of comfort, treatment efficacy, and ongoing treatment side effects based on feedback from both the sleep physician and the patient. Currently, this is time‐consuming as assessment of treatment efficacy in oral appliance requires a sleep study with an oral appliance in situ when the patient is at maximum tolerable mandibular advancement. Recently, simplified home‐based sleep monitoring devices based on mandibular jaw movement with automated analytical algorithms have been suggested as potential alternatives for monitoring the efficacy of oral appliance therapies.^97^ With developments in cloud‐based monitoring technologies incorporated into PAP machines and potentially into oral appliances,^98^ the dentist‐sleep physician partnership and patient monitoring can be further optimized. For example, patient data on oral appliance usage uploaded to a cloud‐based platform can be accessible to both dentists and sleep physicians, allowing monitoring at treatment initiation and during ongoing follow‐up. Moreover, the possibility of patients gaining daily feedback on therapy effectiveness with an oral appliance might lead to more prompt intervention in cases of low compliance/effectiveness and overall improved treatment adherence as evidenced with PAP therapy.

Screening and diagnosis of OSA patients will need to account for patient risk factors, symptoms, comorbidities, and phenotypic characteristics. Implementation of therapy for OSA will need to incorporate the patient's choice of therapy, associated predictors of success for selected therapies, multi‐disciplinary care involving other specialities, and consideration of personalized adjunctive therapy. Ongoing management of OSA therapy will require effective monitoring of patients' therapy and appropriate treatment feedback to patients.

## SUMMARY

There is growing awareness that OSA is a very common disorder that is heterogeneous in terms of risk factors, symptoms, pathophysiology, comorbidity risk, and treatment response. Better characterization of OSA phenotypes is an essential step towards precision medicine approaches that provide accurate diagnoses and more effective treatment options. The tools required to quantify the many pathophysiological phenotypes of OSA and to temporally monitor the presence and severity of OSA over time and in response to therapy are evolving. These new digital tools are supporting the transition to a more personalized precision medicine approach for OSA and promoting more patient engagement. The growing adoption of oral appliance therapy as a first‐line treatment highlights the importance of multi‐disciplinary OSA care, specifically between dentists and sleep physicians. This shift in clinical practice is of high relevance to dentists, who can play a pivotal role in the diagnosis and management of OSA.

## DISCLOSURES

GMS has no conflicts of interest to disclose; BKT has no conflicts of interest to disclose; PAC has an appointment as an endowed Academic Chair at the University of Sydney that was created from ResMed funding; he receives no personal fees, and this relationship is managed by an Oversight Committee of the University. Additionally, he has received research support from ResMed, SomnoMed, and is a consultant/adviser to SomnoMed, ResMed, and Sunrise Medical.

## AUTHOR CONTRIBUTIONS

**GM Stewart:** Conceptualization; writing – original draft; writing – review and editing. **BK Tong:** Conceptualization; writing – original draft; writing – review and editing. **PA Cistulli:** Conceptualization; writing – original draft; writing – review and editing.
